# Study of caveolin-1 gene expression in whole adipose tissue and its subfractions and during differentiation of human adipocytes

**DOI:** 10.1186/1743-7075-7-20

**Published:** 2010-03-12

**Authors:** José M Fernández-Real, Victoria Catalán, José M Moreno-Navarrete, Javier Gómez-Ambrosi, Francisco J Ortega, Jose I Rodriguez-Hermosa, Wifredo Ricart, Gema Frühbeck

**Affiliations:** 1 Service of Diabetes, Endocrinology and Nutrition, Institut d'Investigació Biomèdica de Girona (IdIBGi) Hospital Dr Josep Trueta, 17007 Girona, Spain, CIBEROBN (CB06/03/010) and Instituto de Salud Carlos III (ISCIII) 28029 Madrid, Spain; 2 Department of Endocrinology & Metabolic Research Laboratory, Clínica Universitaria de Navarra 31008 Pamplona, Spain, CIBEROBN (CB06/03/1014) and Instituto de Salud Carlos III (ISCIII) 28029 Madrid, Spain; 3 Department of Surgery, Institut d'Investigació Biomèdica de Girona (IdIBGi) Hospital Dr Josep Trueta,17007 Girona, Spain

## Abstract

**Context:**

Caveolins are 21-24 kDa integral membrane proteins that serve as scaffolds to recruit numerous signaling molecules. Specific subclasses of caveolae carry out specific functions in cell metabolism. In particular, triglycerides are synthesized at the site of fatty acid entry in one of these caveolae classes.

**Objective and Methods:**

We studied the expression of caveolin-1 (*CAV-1*) gene in association with metabolic variables in 90 visceral and 55 subcutaneous adipose tissue samples from subjects with a wide range of fat mass, in the stromovascular fraction (SVC) and isolated adipocytes, and during differentiation of human adipocytes.

**Results:**

*CAV-1 *gene expression was significantly decreased in visceral adipose tissue (v-*CAV-1*) of obese subjects. v-*CAV-1 *was positively associated with several lipogenic genes such as acetyl-coA carboxylase (*ACACA*, r = 0.34, p = 0.004) and *spot-14 *(r = 0.33, p = 0.004). In non-obese subjects v-*CAV-1 *also correlated with fatty acid synthase (*FAS*, r = 0.60, p < 0.0001). Subcutaneous (sc) adipose tissue (s*c-CAV-1*) gene expression was not associated with these lipogenic factors when obese and non-obese subjects were studied together. In obese subjects, however, sc-*CAV-1 *was associated with fatty acid synthase (*FAS*, r = 0.36, p = 0.02), sterol regulatory element binding protein-1c (*SREBP-1c *(r = 0.58, p < 0.0001), *ACACA *(r = 0.33, p = 0.03), *spot-14 *(r = 0.36, p = 0.02), *PPAR-γ co-activator-1 *(*PGC-1*, r = 0.88, n = 19). In these obese subjects, *sc-CAV-1 *was also associated with fasting triglycerides (r = -0.50, p < 0.0001).

*CAV-1 *expression in mature adipocytes was significantly higher than in stromal vascular cells. *CAV-1 *gene expression in adipocytes from subcutaneous adipose tissue (but not in adipocytes from visceral adipose tissue) was significatively associated with fasting triglycerides. *CAV-1 *gene expression did not change significantly during differentiation of human preadipocytes from lean or obese subjects despite significant increase of FAS gene expression.

**Conclusion:**

Decreased *CAV-1 *gene expression was simultaneously linked to increased triglycerides and decreased lipogenic gene expression among obese subjects, paralleling the observations of hypertriglyceridemia in *CAV-1 *knockout mice. However, the regulation of *CAV-1 *gene expression seems independent of the adipogenic program.

## Background

Obesity is an epidemic health problem worldwide that impacts the risk and prognosis of many diseases, including type 2 diabetes mellitus (DM-2), cardiovascular disease, hyperlipidemia, and cancer [1]. Adipose tissue plays a critical role in energy homeostasis in higher organisms. It serves as the main site for energy storage in the form of triglycerides and also contributes to systemic glucose and lipid metabolism via its function as an endocrine organ.

Lipid rafts are lateral assemblies of sphingolipids and cholesterol that function as platforms to which distinct classes of proteins are associated. Caveolae are a specialized type of lipid raft that appear in the plasma membrane as 50-100-nm flask-shaped invaginations that project into the cytosol. Caveolae are present in most cell types but are particularly abundant in adipocytes, where they account for 30% of the plasma membrane surface area [2]. In adipocytes, *CAV-1 *isoform is responsible for caveolae formation [3]. Caveolins are 21-24 kDa integral membrane proteins that serve as scaffolds to recruit numerous signaling molecules. Caveolae function has been implicated in membrane traffic, signal transduction, substrate transport and endocytosis [4]. In recent years, an in-depth knowledge of these functions is increasingly recognized. Specific subclasses of caveolae carry out specific functions in cell metabolism. In particular, triglycerides are synthesized at the site of fatty acid entry in one of these caveolae classes [5].

Fatty acid synthesis is of vital importance in cell physiology in general, and in adipose tissue physiology in particular. The biosynthesis of the fatty acids (lipogenesis) depends on well known enzyme-regulated processes. Studies in humans fed with a high carbohydrate diet demonstrated that total body fat synthesis significantly exceeded hepatic *de novo *lipogenesis, suggesting that adipose tissue may be the major site for fat synthesis, with the adipose tissue accounting for >40% of whole-body lipogenesis [6,7].

Several lines of evidence suggest interplay between *CAV-1 *and fatty acid metabolism. *CAV-1 *null mice had a lean phenotype and their adipocytes lack caveolae, a derangement that translates into a systemic inability for lipid accumulation with increasing age [8]. *CAV-1*^-/- ^mice were hypertriglyceridemic. The impaired triglyceride clearance took place in the setting of normal total and hepatic lipoprotein lipase activity, and reduced hepatic very low-density lipoprotein (VLDL) secretion [9].

Additionally, *CAV-1 *deficiency was associated with an increase in high-density lipoprotein (HDL). *CAV-1 *deficiency prevented the transcytosis of LDL across endothelial cells, and therefore, *CAV-1 *might be implicated in the regulation of plasma LDL levels [9]. These metabolic alterations were not accompanied by noticeable changes in circulating glucose or insulin [8]. In humans, *CAV-1 *is one of the locis identified associated with inherited lipodystrophy and hypertriglyceridemia [10]. In addition, the gene encoding *CAV-1 *has been proposed as a quantitative trait locus of triglyceridemia [11].

We aimed to evaluate the interrelationships among the adipose tissue expressions of *CAV-1*, lipogenic genes and circulating triglycerides in a cohort of obese and non-obese subjects.

## Methods

### Subjects

Ninety adipose tissue samples were obtained from visceral depots during elective surgical procedures (cholecystectomy, surgery of abdominal hernia and gastric by-pass surgery), washed, fragmented and immediately flash-frozen in liquid nitrogen before be stored at -80°C. These at samples were provided from a group of 90 subjects (31 men and 59 women) with a body mass index (BMI) between 21.5 and 70.0 kg/m^2 ^who were invited to participate at the Endocrinology Service of the *Hospital Universitari de Girona Dr. Josep Trueta *(Girona, Spain). Subcutaneous adipose tissue was also obtained from 55 of these subjects, whose characteristics did not differ significantly from the remaining subjects. Fewer subjects were utilized for gene expression in the subcutaneous depot because of tissue availability. All subjects were of Caucasian origin and reported that their body weight had been stable for at least three months before the study. They had no systemic disease other than type 2 diabetes and obesity and all were free of any infections in the previous month before the study. Liver disease and thyroid dysfunction were specifically excluded by biochemical work-up. Other exclusion criteria for those patients included the following: *1*) clinically significant hepatic, neurological, or other major systemic disease, including malignancy; *2*) history of drug or alcohol abuse, defined as >80 g/day, or serum transaminase activity more than twice the upper limit of normal; *3*) an elevated serum creatinine concentration; *4*) acute major cardiovascular event in the previous 6 months; *5*) acute illnesses and current evidence of high grade chronic inflammatory or infective diseases; and *6*) mental illness rendering the subjects unable to understand the nature, scope, and possible consequences of the study. All subjects gave written informed consent after the purpose of the study was explained to them. The institutional review board of each institution approved the protocol.

### Anthropometric measurements

BMI was calculated as weight (in kilograms) divided by height (in meters) squared.

### Analytical determinations

The serum glucose levels were measured in duplicate by the glucose oxidase method with a *Beckman Glucose Analyzer 2 *(*Brea, CA*). The coefficient of variation (CV) was 1.9%. Total serum cholesterol was measured through the reaction of cholesterol esterase/oxidase/peroxidase, using a *BM/Hitachi 747*. HDL cholesterol was quantified after precipitation with polyethylene glycol at room temperature. Total serum triglycerides were measured through the reaction of glycerol-phosphate-oxidase and peroxidase by routine laboratory tests. HbA1c was measured by the high-performance liquid chromatography method (Bio-Rad, Muenchen, Germany). Intraassay and interassay coefficients of variation was less than 4%.

### Isolation of adipose tissue fractions

In a subsample of these subjects, we performed the isolation of adipocyte and stromo-vascular fraction (SVF). Tissues were washed three to four times with phosphate-buffered saline (PBS) and suspended in an equal volume of PBS supplemented with 1% bovine serum and 0.1% collagenase type I (fabricant) prewarmed to 37°C. The tissue was placed in a shaking water bath at 37°C with continuous agitation for 60 minutes and centrifuged for 5 minutes at 300 to 500 g at room temperature. The supernatant, containing mature adipocytes, was recollected. The pellet was identified as the SVF. The adipose tissue fractionation was performed from 10 visceral and 8 subcutaneous fat depots.

#### Cell culture

Commercially available cryo-preserved human subcutaneous pre-adipocytes from two non-diabetic male subjects with age>40 and Body Mass Index (BMI) <25 or BMI>30 Kg/m^2 ^(SP-F-1 or SP-F-3, respectively; *Zen-Bio, Inc.*) were plated on T-75 cell culture flasks and cultured at 37°C and 5% CO_2 _in Dulbecco's modified Eagle's medium (DMEM)/Nutrient Mix F-12 medium (1:1, v/v) supplemented with Fetal Bovine Serum (FBS) 10%, HEPES 1%, Glutamine 1% and Penicillin/Streptomycin (P/S) at 10 U/mL (all from *GIBCO*, BRL; *Grand Island, NY*). One week later, human subcutaneous pre-adipocytes were resuspended and cultured (~40.000 cells/cm^2^, 3^rd ^passage) in 12- or 96-well plates with Pre-adipocyte Medium (PM; *Zen-Bio, Inc.*) composed of DMEM/Nutrient Mix F-12 medium (1:1, v/v), FBS 10%, HEPES 1%, Glutamine 1% and P/S 1% in a humidified 37°C incubator with 5% CO2. Twenty-four hours after plating, cells were checked for complete confluence (day 0) and differentiation was induced using Differentiation Medium (DM; *Zen-Bio, Inc.*), composed of PM with human Insulin (Ins), Dexamethasone (DXM), Isobutylmethyl-xanthine (IBMX) and PPARγ agonists (Rosiglitazone, Rs). After 7 days (day 7), DM was replaced with fresh Adipocyte Medium (AM; *Zen-Bio Inc.*), composed of DMEM/Nutrient Mix F-12 medium (1:1, v/v), FBS, HEPES, Biotin, Panthothenate, human Insulin (Ins), Dexamethasone (DXM), Penicillin, Streptomycin and Amphotericin, according to manufacturers' guidelines. Two weeks after the initiation of differentiation (day 14), cells appeared rounded with large lipid droplets apparent in the cytoplasm. Cells were then considered mature adipocytes (MAs), harvested and stored at -80°C for RNA extraction to study gene expression levels, or fixed and immunostained (96-well plates). For gene expression analyses, three biological replicates (n = 3) of fat cells from both lean and obese subjects were performed.

### Gene expression analyses

RNA was prepared from adipose tissue fragments using RNeasy Lipid Tissue Mini Kit (*QIAgen, US*). The integrity of each RNA sample was checked by either agarose gel electrophoresis or with an Agilent Bioanalyzer^® ^(*Agilent Technologies, Palo Alto, CA*). Total RNA was quantified by means of spectrophotometer (*GeneQuant, GE Health Care, Piscataway NJ*) or with the bioanalyzer. 3 ug of RNA from each fat sample were then reverse transcribed to cDNA using High Capacity cDNA^® ^Archive Kit (*Applied Biosystems, Darmstadt, Germany*) according to the manufacturer's protocol.

Gene expression was assessed by real time PCR using an ABI Prism^® ^7000 Sequence Detection System (*Applied Biosystems, Darmstadt, Germany*), using TaqMan^® ^technology suitable for relative gene expression quantification. The reaction was performed following manufacturers' protocol in a final volume of 25 μl. The cycle program consisted of an initial denaturing of 10 min at 95°C then 40 cycles of 15 sec denaturizing phase at 92°C and 1 min annealing and extension phase at 60°C. Positive and negative controls were included in all the reactions.

The SybrGreen^® ^primer sets used were previously validated to give an optimal amplification over serial dilutions of target, and analysis of melting curves demonstrated specific single product for each gene primer. Primer sequences were as follows: PPIA forward/reverse primer sequences were 5'-CAAATGCTGGACCCAACACAA/CCTCCACAATATTCATGCCTTCTT-3' respectively; *CAV-1 *forward/reverse primer sequences were 5'-AACGATGACGTGGTCAAGATTG-3' and 5'-TCCAAATGCCGTCAAAACTGT-3', respectively.

The commercially available and pre-validated TaqMan^® ^primer/probe sets used were as follows: Cyclophilin A (*PPIA; Hs99999904_m1, RefSeq. NM_002046.3*) was used such as endogenous control for all target genes in each reaction and Spot 14 homolog rat (*THRSP; Hs009300 58_m1, RefSeq. NM_003251.2*), Fatty Acid Synthase (*FASN; Hs00188012_m1, RefSeq. NM_004104.4*), Acetyl-Coenzyme A Carboxylase alpha (*ACACA; Hs00167385_m1, RefSeqs. NM_198834.1, NM_198836.1, NM_19883 7.1, NM_198838.1 and NM_198839.1*) and Sterol regulatory element binding protein 1 (*SREBP1; Hs00231674_m1, RefSeq. NM_004104.4*) were the target genes.

In both RT-PCR techniques, SybrGreen^® ^and TaqMan^®^, a threshold cycle (Ct value) was obtained for each amplification curve and a ΔCt value was first calculated by subtracting the Ct value for human Cyclophilin A (PPIA) cDNA from the Ct value for each sample and transcript. Fold changes compared with the endogenous control were then determined by calculating 2^-ΔCt^, so gene expression results are expressed in all cases as expression ratio relative to PPIA gene expression according to manufacturers' instructions.

### Western blot

Tissues were homogenized and protein content was measured as follows. Equal amounts of protein (25 μg) were run out in 12% SDS-PAGE, subsequently transferred to nitrocellulose membranes (Bio-Rad Laboratories, Inc., Hercules, CA) and blocked in Tris-buffered saline (10 mmol/L Tris-HCl, 150 mmol/L NaCl, pH 8.0) with 0.05% Tween 20 (TBS-T) containing 5% non-fat dry milk for 1 h at room temperature (RT). Blots were then incubated overnight at 4 °C with a rabbit polyclonal anti-CAV1 antibody (C 4490, Sigma, St Louis, MO) or murine monoclonal anti-β-actin (A 5441, Sigma). The antigen-antibody complexes were visualized using horseradish peroxidase-conjugated anti-rabbit or anti-mouse IgG antibodies (1:5,000) and the enhanced chemiluminescence ECL detection system (Amersham Biosciences, Buckinghamshire, UK). The intensity of the bands was determined by densitometric analysis with the Gel Doc™ gel documentation system and Quantity One 4.5.0 software (Bio-Rad) and normalized with β-actin densitometric values. All assays were performed in duplicate.

### Statistical analyses

Descriptive results of continuous variables are expressed as mean ± SD. Before statistical analysis, normal distribution and homogeneity of the variances were evaluated using *Levene's *test. Paired and unpaired t-tests, or one-way ANOVA for multiple comparisons, using post-hoc by *Bonferroni's *test when equal variances could be assumed, was used to compare groups with respect to continuous variables. Relation between quantitative variables was tested using *Pearson's *test. All data were expressed as means ± SD. The statistical analyses and graphics were performed using the program SPSS (version 13.0).

## Results

**Figure 1:**
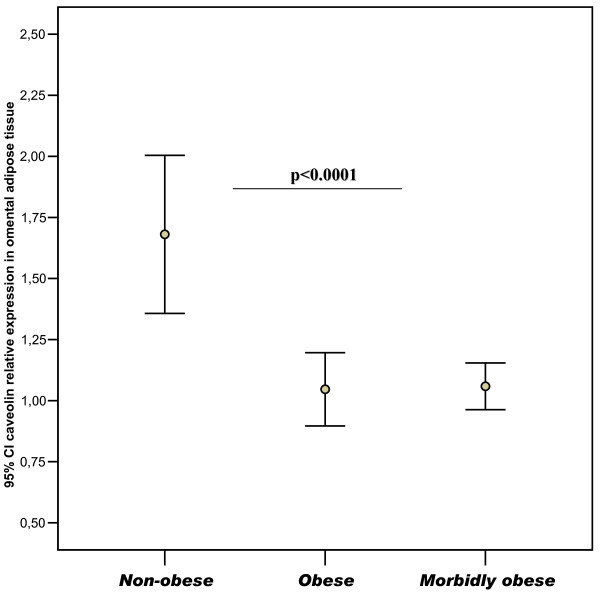
**Gene expression levels for visceral-CAV-1**. Mean and 95% confidence interval for the mean of CAV-1 levels in visceral adipose tissue from non-obese (BMI<30 kg/m^2^), obese (30 ≤ BMI<40 kg/m^2^) and morbidly obese subjects (BMI ≥ 40 kg/m^2^). The differences between obese and non-obese groups were highly significant (p < 0.0001).

**Figure 2:**
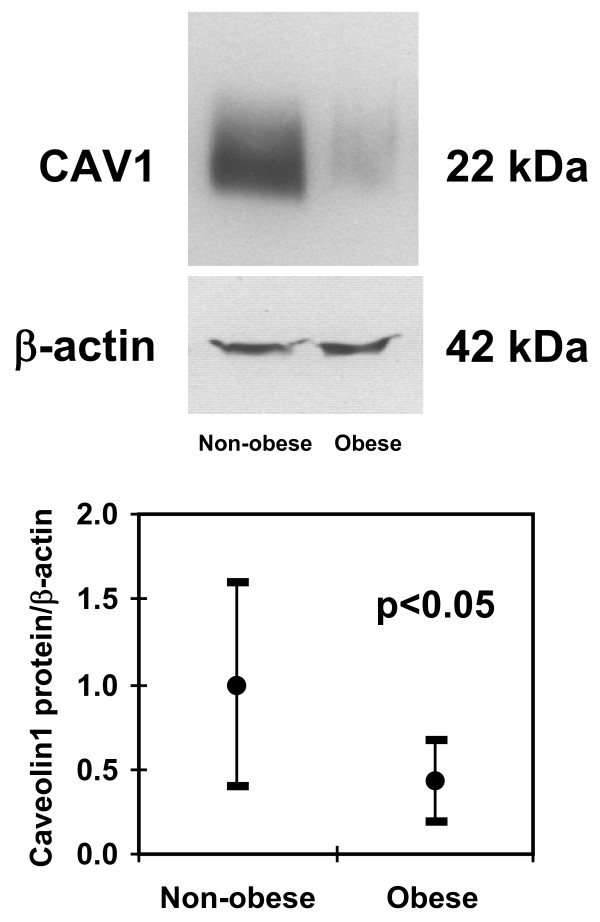
**Western blot**. Protein validation showing decreased CAV-1 protein levels in obese subjects, and the 95% confidence interval for the mean of relative CAV-1 levels in non-obese and obese subjects (lower panel).

The anthropometric and metabolic variables of the studied subjects and the gene expression levels for *CAV-1*, *FAS*, sterol-regulatory-element-binding protein-1c (*SREBP-1c*) and *Spot-14 *from both men and women are summarized in Table 1. Of the studied subjects, 55 had normal fasting glucose, 23 impaired fasting glucose and 12 type 2 diabetes. Obese subjects showed decreased expression of *CAV-1 *in visceral but not in subcutaneous adipose tissue (Table 1 and Figure 1). These results were confirmed at the protein level (Figure 2).

**Table 1:** Anthropometric and biochemical characteristics of studied subjects.

	Non-obese	Obese	Morbidly Obese	p
**N**	39	17	34	
**Sex (M/F)**	15/24	6/11	10/24	0.72
**Body Mass Index (kg/m^2^)**	25.4 ± 3.5	35.3 ± 3.1	46.1 ± 5.5	< 0.0001
**Age (years)**	46.7 ± 13.7	53.1 ± 14.9	43.1 ± 11	0.03
**Glucose (mg/dl)**	93.8 ± 32.2	91.25 ± 11.8	124.8 ± 59.4	0.005
**Cholesterol (mg/dl)**	195.5 ± 41.5	182.2 ± 34.8	189 ± 31.6	0.47
**LDL-cholesterol (mg/dl)**	118 ± 35.8	99.6 ± 32.1	110.8 ± 36.7	0.27
**HDL-cholesterol (mg/dl)**	57.2 ± 16.7	54.5 ± 16.9	59.15 ± 58.7	0.93
**Log fasting tryglicerides**	1.98 ± 0.2	2.03 ± 0.2	2.11 ± 0.2	0.08
**ACACA visceral (RU)**	0.04 ± 0.01	0.017 ± 0.003	0.02 ± 0.001	0.001
ACACA subcutaneous (RU)*	0.033 ± 0.006	0.015 ± 0.002	0.02 ± 0.002	0.03
**FASN visceral (RU)**	0.16 ± 0.02	0.07 ± 0.01	0.09 ± 0.01	0.02
FASN subcutaneous (RU)*	0.36 ± 0.14	0.07 ± 0.02	0.06 ± 0.009	0.01
**SREBP-1c visceral (RU)**	0.018 ± 0.002	0.013 ± 0.001	0.014 ± 0.001	0.2
SREBP-1c subcutaneous (RU)*	0.019 ± 0.004	0.009 ± 0.001	0.009 ± 0.001	0.04
**Spot-14 visceral (RU)**	0.26 ± 0.03	0.26 ± 0.03	0.23 ± 0.01	0.6
Spot-14 subcutaneous (RU)*	0.60 ± 0.05	0.45 ± 0.003	0.48 ± 0.03	0.04
**Caveolin visceral (RU)**	1.68 ± 0.9	1.04 ± 0.3	1.05 ± 0.3	< 0.0001
Caveolin subcutaneous (RU)*	1.50 ± 0.4	1.78 ± 0.8	1.44 ± 0.4	0.22

**ACACA**, acetyl coA carboxylase; **FAS**, fatty acid synthase; **SREBP-1c**, sterol-regulatory-element-binding protein; **RU**, relative units. Values are expressed as mean ± SD except for gene expressions that are expressed as mean ± SEM.

*Subcutaneous adipose samples were studied in 16 non-obese, 13 obese and 26 morbidly obese subjects.

### Associations of CAV-1 expression with lipogenic genes

**Figure 3:**
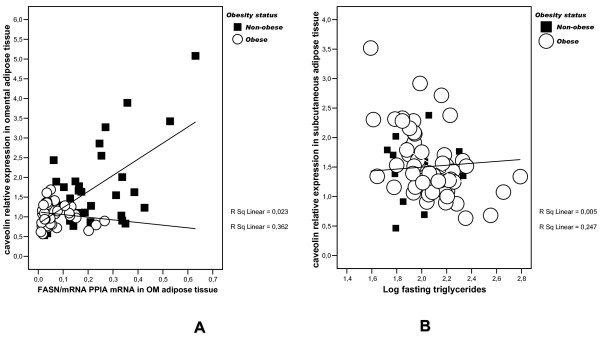
**Linear relationships**. Visceral adipose tissue *CAV-1 *gene expression correlated significantly with the main lipogenic genes (fatty acid synthase is depicted here) in non-obese but not among obese subjects. **B**. Linear relationship between subcutaneous *CAV-1 *gene expression and fasting triglycerides in obese and non-obese subjects.

In all subjects as a whole, *CAV-1 *gene expression in visceral adipose tissue (v-*CAV-1*) was positively associated with the lipogenic genes *acetyl-coA carboxylase *(*ACACA*, r = 0.34, p = 0.004), *Spot-14 *(r = 0.33, p = 0.004), *PPAR-γ co-activator-1 (PGC-1) *(r = 0.57, p = 0.003, n = 24) and tended to be associated with *FAS *gene expression (r = 0.21, p = 0.07). v-*CAV-1 *was not associated with *SREBP-1c *gene expression (r = -0.08, p = 0.4). However, these associations were mainly due to the observations in non-obese subjects, in whom v-*CAV-1 *correlated with *FAS *(r = 0.60, p < 0.0001, Figure 3A), *Spot-14 *(r = 0.35, p = 0.006) and *PGC-1 *(r = 0.93, p = 0.002, n = 7) gene expressions. All these associations were not significant in obese subjects. Fasting triglycerides correlated weakly with v-*CAV-1 *(r = -0.26, p = 0.049).

Divergent results were observed regarding *CAV-1 *gene expression in subcutaneous adipose tissue (sc-*CAV-1*), which was only associated with PGC-1 gene expression (r = 0.47, p = 0.009, n = 30) when obese and non-obese subjects were studied together. In obese subjects, sc-*CAV-1 *was associated with *FAS *gene expression (r = 0.36, p = 0.02), *ACACA *(r = 0.33, p = 0.03), *SREBP-1c *(r = 0.58, p < 0.0001), *spot-14 *(r = 0.36, p = 0.02), *PGC-1 *(r = 0.88, p = 0.001, n = 19). None of these associations was significant in non-obese subjects.

### Associations with metabolic variables

Fasting glucose or glycated hemoglobin were not significantly associated with v-*CAV-1 *or sc-*CAV-1*. However, in non-obese subjects with normal fasting glucose, sc-*CAV-1 *was negatively associated with glycated hemoglobin (r = -0.58, p = 0.004, n = 12).

In obese subjects, sc-*CAV-1 *was associated negatively with fasting triglycerides (r = -0.50, p < 0.0001, Figure 3B). Multiple linear regression analyses disclosed that age (p = 0.027), fasting glucose (p = 0.002) and sc-*CAV-1 *gene expression (p = 0.002) contributed to 8%, 12% and 20%, respectively, of fasting triglycerides variance after adjusting for BMI and sex.

### Study of CAV-1 expression in tissue fractions and during differentiation of preadipocytes

**Figure 4:**
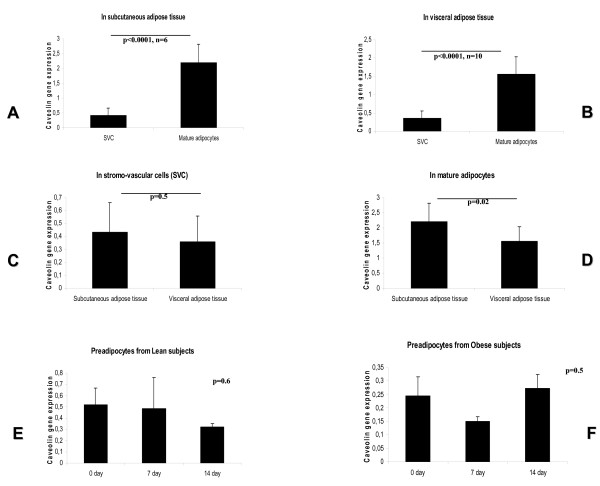
**Cav-1 gene expression levels in isolated fat cell and during differentiation**. Comparison of *CAV-1 *gene expression between isolated stromovascular cells (SVC) and mature adipocytes in **A**. Subcutaneous adipose tissue and **B**. Visceral adipose tissue. **C**. Comparison of *CAV-1 *gene expression in SVC between subcutaneous and visceral adipose tissue. **D**. Comparison of *CAV-1 *gene expression in mature adipocytes between subcutaneous and visceral adipose tissue. **E**. Study of *CAV-1 *gene expression during differentiation of preadipocytes from lean subjects (n = 3). **F**. Study of *CAV-1 *gene expression during differentiation of preadipocytes from obese subjects (n = 3).

In both subcutaneous and visceral fat depots, *CAV-1 *expression in mature adipocytes was significantly higher than in SVF (Figure 4A and 4B). In the visceral but not in the subcutaneous fat depot, *CAV-1 *expression in adipocytes was significantly higher than in SVF (1.56 ± 0.47 *vs. *0.36 ± 0.19, p < 0.0001, Figure 4C and 4D). *CAV-1 *gene expression in adipocytes from subcutaneous adipose tissue (but not in adipocytes from visceral adipose tissue) was significantly associated with fasting triglycerides (r = -0.95, p < 0.0001). Conversely, *CAV-1 *expression in SVF from visceral adipose tissue was negatively associated with fasting glucose (r = -0.77, p = 0.01), glycated hemoglobin (r = -0.87, p = 0.01) and fasting triglycerides (r = -0.81, p = 0.01).

Finally, *CAV-1 *gene expression did not change significantly during differentiation of human preadipocytes from lean or obese subjects (Figure 4D and 4E) despite significant increase of *FAS *gene expression.

## Discussion

The main findings of this manuscript are: 1) *CAV-1 *gene expression was significantly decreased in visceral adipose tissue of obese subjects (Figure 1); in fact, this is the first large study evaluating the expression of *CAV-1 *in adipose tissue. CAV-1 gene expression has been previously reported in a sample of 15 subjects [12]. 2) *CAV-1 *gene expression in whole adipose tissue was significantly associated with the expression of lipogenic genes and fasting triglycerides; 3) *CAV-1 *gene expression was significantly increased in mature adipocytes compared with SVC fraction from both visceral and subcutaneous adipose tissue (Figure 3A and 3B); 4) the expression of *CAV-1 *was significantly decreased in mature adipocytes from the visceral fat compartment; and 5) *CAV-1 *gene expression did not change significantly during differentiation of human preadipocytes from lean or obese subjects.

The type of fat depot (subcutaneous vs. visceral) and obesity status significantly influenced these associations. In non-obese subjects, with a wide range of *CAV-1 *in visceral adipose tissue (v-*CAV-1*), significant and positive associations of v-*CAV-1 *with the main lipogenic genes were observed. Decreased expression of v-*CAV-1 *of obese subjects, with a relatively low range of v-*CAV-1*, could have led to the absence of relationship with lipogenic genes in this obese group. *CAV-1 *gene expression is known to be controlled by sterol-regulatory-element-binding protein (SREBP) [4], one of the main transcriptional factors involved in lipogenesis, so these findings should be interpreted in this context.

Fasting triglycerides were not linked to v-*CAV-1 *in obese and non-obese subjects. sc-*CAV-1*, however, was associated with lipogenic genes (*FAS, ACACA, SREBP-1c, PGC-1 *and *Spot-14 *[13]) and fasting triglycerides only in obese subjects. Those obese subjects with decreased sc-*CAV-1 *had the highest fasting triglyceride concentrations, paralleling, to some extent, the observations of hypertriglyceridemia in *CAV-1 *knockout mice [8]. The question remains why only decreased sc-*CAV-1 *and not decreased v-*CAV-1 *was linked to increased triglycerides. In fact, we found that *CAV-1 *gene expression in adipocytes was significantly higher than in SVF in both subcutaneous and visceral fat depots. Even though, when we compared adipocyte fractions, only *CAV-1 *expression in subcutaneous adipose tissue was negatively associated with fasting triglycerides. In this sense, the protective "metabolic" role of subcutaneous adipose tissue is increasingly recognized [14]. *CAV-1 *expression in subcutaneous adipose tissue could be the reflection of a protective role of *CAV-1 *in the metabolism of triglycerides.

CAV-1 gene expression did not change significantly during differentiation of human pre-adipocytes despite a significant increase of FAS gene expression, a marker of adipocyte differentiation. This is in contrast to what has been observed in 3T3-L1 cells, in which CAV-1 gene expression increases during differentiation [15-17]. This finding suggests that *CAV-1 *is not under the control of adipogenic factors in human adipocytes but influenced by other components located in the microenvironment of the adipose tissue. For instance, *CAV*-1 gene expression has been detected in macrophages under the influence of lipopolysaccharide [18,19], a bacterial product known to be increased in human obesity [20,21]. However, we cannot exclude that human pre-adipocytes have already entered into senescence. In fact, increased caveolin-1 has been described as a cause for the declined adipogenic potential of senescent human mesenchymal stem cells[22].

On the other hand, the inverse association between *CAV-1 *gene expression and fasting glucose, glycated hemoglobin and fasting triglycerides in SVC from visceral fat could reflect the differential metabolic activity of the visceral fat depot.

Decreased expression of v-*CAV-1 *is in contrast with previous findings in women, in whom both v-*CAV-1 *and sc-*CAV-1 *were increased among obese women [12]. However, this latter study was performed in young women. Estrogen receptors have been reported to associate with and regulate the production of *CAV-1 *[23,24]. It is thus possible that the confounding effects of estrogens in pre-menopausal women could have played a role in these discrepant results.

A remarkable reduction in the expression of the genes controlling lipogenic enzymes, namely SREBP-1c, and genes coding lipogenic enzymes (*FAS*, ***phosphoenol pyruvate carboxykinase ****(PEPCK), ATP citrate-lyase, pyruvate carboxylase*) [25-27] has been demonstrated in obesity, a situation in which hypertriglyceridemia is frequently present despite that triglyceride synthesis and lipogenic genes are positively correlated. In *CAV-1 *null mice the impaired triglyceride clearance took place in the setting of normal total and hepatic lipoprotein lipase activity, and reduced hepatic very low-density lipoprotein (VLDL) secretion[9].

## Conclusions

Caveolins are integral membrane proteins that serve as scaffolds to recruit numerous signaling molecules, implicated in membrane traffic, signal transduction, substrate transport and endocytosis. The finding of decreased *CAV-1 *gene expression in adipocytes from obese subjects, and the associations with lipogenic genes and triglycerides will help to understand the alterations of intracellular signaling and lipid synthesis in obesity. The regulation of *CAV-1 *gene expression seems independent of the adipogenic program.

## Competing interests

The authors (JMF-R; VC; JMM-N; JG-A; FJO; JIR-H; WR; GF) report no competing interests.

## Authors' contributions

JMF-R participates in the analysis and interpretation of data and drafted the manuscript. JMM-N, VC, JIR-H and FJO carried out the biochemical analyses, participates in acquisition of data and in the biochemical and clinical determinations, and reviewed the manuscript., JG-A: Participate in design and reviewed the manuscript critically for important intellectual content. WR and GF: Participate in design and coordination, and helped to draft the manuscript and reviewed the manuscript critically for important intellectual content. All authors read and approved the final manuscript.
